# T179. DO INDIVIDUALS IN A CLINICAL HIGH-RISK STATE FOR PSYCHOSIS DIFFER FROM HEALTHY CONTROLS IN THEIR CORTICAL FOLDING PATTERNS?

**DOI:** 10.1093/schbul/sby016.455

**Published:** 2018-04-01

**Authors:** Rachele Sanfelici, Dominic B Dwyer, Anne Ruef, Linda A Antonucci, Nikolaos Koutsouleris, PRONIA Study Group

**Affiliations:** 1 Ludwig Maximilian University; 2 University of Bari “Aldo Moro”

## Abstract

**Background:**

Volumetric brain differences between persons meeting criteria for a clinical high-risk state for psychosis (CHR) and healthy controls (HC) have been previously reported, yet little is known about potential abnormalities in surface-based morphological measures. Gyrification (i.e., the amount of cortical convolution) remains relatively stable across the lifespan and is minimally influenced by ubiquitous confounding factors (e.g., drug use, medication, or stress). Recently, a multi-site analysis conducted in 104 CHR persons found global increases in cortical gyrification compared to HC (Sasabayashi et al. 2017). If replicated, gyrification abnormalities in CHR could potentially serve as early neuromarkers of elevated risk, and thus could eventually be used to identify objectively and efficiently the CHR state.

**Methods:**

A total of 124 CHR and 264 HC subjects were recruited as part of the PRONIA consortium (www.pronia.eu), a large-scale international longitudinal study currently consisting of 10 European sites. Cortical surfaces were reconstructed from structural MRI images using a volume-based, newly introduced technique called the Projection-Based-Thickness (PBT) as available in the SPM-based-toolbox CAT12. Local gyrification was quantified automatically across the whole brain as absolute mean curvature for each vertex of the brain surface mesh consisting of thousands of individual measurement points. Vertex-wise differences of curvature values were calculated applying a General Linear Model, corrected for age, gender and site effects. Results were investigated at corrected and uncorrected levels.

**Results:**

We found no significant differences in vertex-wise gyrification between CHR and HC at either corrected or uncorrected levels (p>0.05). Further investigations of potential confounding site effects also did not reveal differences.

**Discussion:**

Our preliminary findings suggest that CHR subjects do not show whole-brain gyrification abnormalities when compared with healthy subjects. These negative results agree with literature suggesting that cortical convolution might be more affected by neurodevelopmental or genetic factors, and thus deviations from normal patterns might not be detectable in heterogeneous samples of at-risk subjects wherein the etiology and ultimate prognosis is unknown. In order to better investigate differences in cortical folding and address the role of gyrification as neuroanatomical biomarker for psychosis, future investigations should focus on subgroups within CHR populations (e.g. patients groups defined by basic symptoms, ultra-high risk, or familial risk) in addition to specific analyses of individuals with higher neurodevelopmental (e.g., obstetric complications) or genetic (e.g., polygenic risk) loadings.

